# From period poverty to policy change: advancing menstrual health as a public health priority in Nigeria

**DOI:** 10.3389/frph.2025.1686031

**Published:** 2025-10-16

**Authors:** Francisca Ogochukwu Onukansi

**Affiliations:** ^1^ Department of Public Health, Federal University of Technology Owerri, Owerri, Nigeria; ^2^ Youth in Research Hub, Enugu, Nigeria

**Keywords:** menstrual health, period poverty, gender equality, public health policy, Nigeria

## Abstract

Period poverty, the lack of access to menstrual products, adequate sanitation, and reliable health information, continues to undermine the health, dignity, and opportunities of millions of girls and women in Nigeria. Closely linked to gender inequality, the problem is compounded by stigma, inadequate infrastructure, and economic barriers, especially for low-income and marginalized groups. While civil society initiatives and isolated government gestures exist, Nigeria still lacks a comprehensive national menstrual health policy. In contrast, countries such as Scotland, Kenya, and Colombia have advanced reforms through product subsidies, menstrual education, and integrated WASH systems. Drawing on these global experiences and local efforts by Nigerian organizations, this commentary calls for a coordinated, government-led response that embeds menstrual health into public health, education, and social protection frameworks. Achieving menstrual equity will require sustained political commitment, structural investment, and culturally responsive policies.

## Introduction

Each day, roughly 800 million women and girls globally, are menstruating; yet almost one-third of them lack the clean water, safe and private sanitation facilities, hygiene infrastructure, and suitable menstrual products required to manage their periods with dignity (1, 2). The absence of these essentials does more than interrupt daily routines, it infringes on basic human rights, constrains educational and economic prospects, and compounds gender inequities.

Menstrual-health management entails not only access to clean, absorbent materials but also the availability of secure, private, and dignified spaces in which those materials can be used effectively (3). When these conditions are unmet, the result is period poverty, a multidimensional problem rooted in economic, social, cultural, and political barriers (4). Globally, of the estimated 1.9 billion people who menstruate, nearly 500 million cannot manage their periods adequately (5).

The burden is especially acute in sub-Saharan Africa, where one in ten girls is absent from school due to menstruation, and up to 20% eventually drop out for reasons directly or indirectly linked to menstruation (1, 5). In Nigeria, more than half of school-age girls stay home each month for fear of staining their uniforms and facing ridicule because sanitary products are unaffordable or unavailable (6). Nearly one in four Nigerian children also lack access to private sanitation facilities for toileting and menstrual hygiene, a gap that has contributed to school dropout for an estimated 10 million children, the majority of whom are girls. Period poverty is widespread among adolescents and young women: a recent survey in a low-income community in Lagos found a 50% prevalence, with many forced to use unhygienic substitutes such as newspapers, old sanitary towels, rags, tissue, or scraps of cloth. Such practices heighten the risk of reproductive and urinary tract infections while inflicting emotional distress that undermines school attendance and self-esteem (7, 8, 9). Economic dependence further drives some girls to engage in transactional sex to obtain menstrual supplies, increasing their vulnerability to gender-based violence, HIV, and other infections (6). Cultural norms compound these risks: in certain communities, menstruation signals marriage readiness, yet remains taboo to discuss, even between mothers and daughters (10).

Recognizing its far-reaching repercussions, the United Nations has linked period poverty to six Sustainable Development Goals, SDGs 1, 3, 4, 5, 6, and 10, underscoring that equitable menstrual health is integral to poverty reduction, health, education, gender equality, water and sanitation, and the reduction of inequalities (2). Addressing period poverty therefore demands multi-sectoral investment, rigorous policy attention, and the dismantling of menstrual stigma so that the biological fact of menstruation no longer limits the rights, freedoms, and potential of those who experience it as highlighted in Hennegan et al. (11), findings. Despite growing recognition of menstrual health as a human right, Nigeria lacks a comprehensive national policy framework to address period poverty at scale. Therefore, this paper explores the intersection of menstrual health and public policy in Nigeria, analyzing the current gaps and opportunities for systemic change. It advocates for prioritizing menstrual health as a fundamental public health issue, critical for achieving gender equality, educational equity, and national development and drawing insight from successful global policy.

### Current landscape: interventions and observed gaps in Nigeria

Globally, an expanding alliance of researchers, donors, NGOs, UN agencies, women-led grassroots organizations, major feminine hygiene brands, and social entrepreneurs is working to raise awareness and channel resources toward addressing the shame, stigma, and taboos surrounding menstruation, especially among girls in low- and middle-income countries (LMICs) (12). Notably, Nigeria's response to period poverty has expanded markedly since 2020, yet the overall effort remains fragmented and heavily NGO-driven. At the policy level, the only nationwide measure to date is the 2020 Finance Act VAT exemption on locally-manufactured sanitary pads (13). While hailed as a breakthrough, subsequent market analyses show that the waiver has had little effect on retail prices because production inputs and supply-chain tariffs were left untouched, muting affordability gains for low-income women (13, 14). Beyond this limited fiscal step, menstrual health is still absent from federal health or education statutes, and no dedicated budget line exists for menstrual-health commodities or WASH facilities in schools.

Humanitarian and multilateral agencies have tried to fill this vacuum, particularly in conflict-affected states such as in Nigeria. UNFPA's adolescent-girls programme in Borno State delivers dignity kits and school-based sensitisation; in 2022 alone, the agency reached 120 girls (and seven boys as peer supporters) in Maiduguri's G.R.A. Model School, pairing distribution with stigma-reduction sessions (15). These efforts illustrate how menstrual health is folded into broader gender-based-violence and displacement responses, but coverage remains episodic and resource-dependent. Additionally, International WASH actors have integrated menstrual hygiene management into school health promotion. WaterAid Nigeria's 2023 campaign, funded by Kimberly-Clark, trained 80 teachers and over 180 students across 39 Lagos schools, adding reusable-pad demonstrations to its curriculum (16).

In Southeastern Nigeria, our grassroot organizations, Like the HopeForHer Humanitarian Foundation, which I co-founded establishing PadBanks intatives in Rural secondary schools with a plastic recycling for sustainability plan. The Padbank Initiative donated over 2000 sanitary pads to rural schools (17). Similarly, in Northern Nigeria, the Five Cowries' Hubs in collaboration with the Nigeria Health Watch, launched the Art Based Menstrual health education, which has helped create awareness and promote menstrual health (18). Additionally, Youth and private-sector hybrids are also emerging. For Instance, Avon Healthcare in Collaboration with Safety for Every Girl, tied a social-media campaign to donations of reusable kits for 500 girls in Lagos (19). Also, the Run Club Abuja and the AWP Network organised a 10 km charity run that financed six-month pad supplies for 500 students (20).

Collectively, these initiatives have normalised public discussion of menstruation and piloted alternative distribution models such as pad banks, reusable-pad training, art-based education etc. Nevertheless, their limitations are consistent: short funding cycles, duplication of effort, inadequate monitoring data, and minimal engagement with curricular reform. As a result, the average rural secondary-school girl still lacks reliable access to dignified WASH facilities and affordable products, underscoring the need for statutory backing and pooled financing.

### Why policy change is critical

Policy change is a vital lever for eliminating period poverty, as demonstrated by successful global reforms (21) and advocacy efforts led by women and girls have catalyzed legal and fiscal changes in several countries. In 2020, Scotland passed the Period Products (Free Provision) Act, which became operative in August 2022, making Scotland the first country in the world to provide free period products for all who need them (21). Furthermore, this evidence suggests that free pad distribution leads to significantly improved access, particularly among low-income populations (21). This ground-breaking policy provides a model for countries like Nigeria where period poverty is widespread and systemic.

European countries have similarly made strategic policy moves to reduce economic barriers to menstrual health. Germany, for instance, lowered VAT on menstrual products from 17% to 7% in 2020, reclassifying these items as essential goods rather than luxury items (21). More than 17 European countries have now reduced or eliminated VAT on menstrual products altogether (6, 21). These fiscal policies demonstrate how governments can directly reduce affordability challenges through targeted taxation reforms.

Outside Europe, advocacy and popular movements have driven important policy changes. In Jamaica, menstrual products were added to the list of items exempt from the General Consumption Tax, making it the first Caribbean country to abolish the so-called “tampon tax” (22). Similarly, in Canada, the highly successful #NoTaxOnTampons campaign led to the removal of the 5.0% federal tax on menstrual products in 2015, reversing an earlier failed attempt from 2004 (22). In Colombia, the grassroots movement #MenstruacionLibreDeImpuestos led to a 2018 Supreme Court decision eliminating taxes on menstrual products, with further expansions in 2020 to include menstrual cups and period underwear (22). These examples highlight how grassroots campaigns, when persistent and well-organized, can influence national policy and shift menstruation from a private concern to a matter of public justice.

African countries have also demonstrated leadership in menstrual policy reform. Kenya took early action by removing taxes on menstrual products in 2004 and, by 2017, began distributing free sanitary pads in public schools (23). South Africa followed in 2019 by supplying free disposable pads to girls from low-income households, while Botswana now provides free pads in both public and private schools (23). These policies are not just symbolic; they have been linked to improved school attendance, reduced stigma, and enhanced gender equity.

In Nigeria, however, efforts remain fragmented and inadequate. The 2020 VAT exemption on locally manufactured sanitary pads was a positive step, signalling initial government recognition of menstrual health as a public issue. Yet its impact was muted due to broader supply chain inefficiencies and the absence of input duty waivers or import relief. As a result, the average market price of menstrual products has remained largely unchanged (14). This shows that VAT reform alone is insufficient; meaningful policy change must be holistic and backed by regulatory oversight and economic instruments (22).

Moreover, despite a growing number of menstrual health initiatives by NGOs and development agencies, effort remains small-scale, donor-funded, and unsustainable in the long term. These programs, while commendable, are not a substitute for state-level intervention. Approximately 37 million Nigerian girls and women are estimated to lack access to adequate menstrual health products and hygiene infrastructure (24). Without a coherent, national policy framework to guide funding, procurement, and implementation, these fragmented efforts will continue to fall short.

Therefore, policy change is not only necessary, it is foundational. Nigeria needs a rights-based, institutional approach that embeds menstrual health within its education, health, and social protection systems. This includes government-financed provision of free or subsidized products in schools and health centers, national education campaigns to combat menstrual stigma, and public budgeting for menstrual health infrastructure. Policy must move beyond symbolic gestures and become a vehicle for real, equitable, and lasting change.

### Recommendations

To effectively tackle period poverty in Nigeria, a coordinated, policy-led approach is essential. The government should prioritize the development and enactment of a comprehensive National Menstrual Health Policy that integrates education, health, WASH (water, sanitation, and hygiene), and gender equality frameworks. Building on existing VAT exemptions, there should be subsidized or free access to menstrual products for schoolgirls, internally displaced persons (IDPs), and low-income women, supported by tax incentives for local manufacturers.

In parallel, menstrual health education must be mainstreamed into national school curricula to reduce stigma and equip both girls and boys with accurate knowledge. Adequate WASH infrastructure in schools and public institutions is also critical to ensuring safe and dignified menstrual hygiene management.

Furthermore, government agencies must invest in menstrual health research and monitoring systems to track policy impact and support health outcomes. A basic monitoring framework could include: (i) product access and affordability, measured through household and market surveys; (ii) adequacy of WASH facilities in schools, captured through national education and WASH assessments; (iii) school absenteeism linked to menstruation, tracked within education sector data systems; and (iv) stigma and knowledge levels, assessed through periodic perception or health surveys. These indicators, aligned with existing national data sources, would provide accountability and guide evidence-based policy refinement.

Ultimately, working with and providing funding support for grassroots organizations such as HopeforHer humanitarian foundation, Resilient Girl Initiative, etc is vital, as these groups offer community-driven solutions that should inform national strategies. Additionally, a sustained public awareness campaign and the appointment of menstrual health policy champions can help shift cultural norms, foster political will, and ensure menstrual health becomes a public health and human rights priority in Nigeria.

At the same time, the risks of large-scale free provision must be anticipated. Without strong systems, leakage and diversion of supplies can undermine access for intended beneficiaries. These risks can be mitigated by integrating distribution into existing school health and primary health centre supply chains, supported by digital stock monitoring. Cultural preferences and stigma also affect uptake; offering both disposable and reusable products, alongside menstrual health education, can improve acceptability. Environmental concerns around disposable pads can be reduced by incentivising local manufacturing of biodegradable options and supporting reusable pad initiatives. Finally, stable financing and private sector partnerships are critical to avoid supply disruptions. By addressing these risks proactively, free provision can remain both equitable and sustainable.

### Limitations

This perspective relies primarily on secondary evidence, including peer-reviewed studies, official reports, and NGO data. While care was taken to prioritize Nigeria-specific statistics were available, some figures draw on global estimates or advocacy sources. The absence of nationally representative primary data is a limitation and highlights the need for further context-specific research.

## Conclusion

Menstrual health is a fundamental public health and human rights issue that remains critically under-prioritized in Nigeria. Despite growing awareness and scattered interventions, millions of girls and women continue to face period poverty, stigma, and inadequate access to safe products and sanitation. Global evidence shows that transformative change is possible when menstrual health is backed by comprehensive policy, political will, and sustained investment. For Nigeria, the path forward must involve enacting robust national policies, removing structural and economic barriers, and centering the voices of women and girls in decision-making. Addressing menstrual health is not just a matter of hygiene, it is essential to achieving gender equity, educational attainment, and national development.

## Data Availability

The original contributions presented in the study are included in the article/Supplementary Material, further inquiries can be directed to the corresponding author.
